# FISH for All: A Fast and Efficient Fluorescent *In situ* Hybridization (FISH) Protocol for Marine Embryos and Larvae

**DOI:** 10.3389/fphys.2022.878062

**Published:** 2022-04-19

**Authors:** Periklis Paganos, Filomena Caccavale, Claudia La Vecchia, Enrico D’Aniello, Salvatore D’Aniello, Maria Ina Arnone

**Affiliations:** Department of Biology and Evolution of Marine Organisms, Stazione Zoologica Anton Dohrn, Naples, Italy

**Keywords:** gene expression, whole-mount FISH, echinoderms, mollusks, tunicates, cephalochordates, development

## Abstract

*In situ* hybridization is one the most commonly used techniques for developmental and evolutionary biology and has extensively contributed to the identification of distinct cell types and cell states, as well dissecting several molecular mechanisms involved in physiological processes. Moreover, it has been used as a tool to compare distinct gene expression patterns and, therefore, genetic programs across animal species. Nowadays, the predominance of transcriptomics in science has imposed the need to establish a reliable, fast and easy whole mount *in situ* hybridization protocol. Here we describe a fluorescent *in situ* hybridization protocol that is rapid, accurate and applicable in a great variety of marine species.

## Introduction

A key element for understanding the development, physiology and evolutionary origins of an organism is gathering information on the cell types they harbor. Cell type identification relies on the characterization of distinct morphological features, often linked to their functionality and the reconstruction of their molecular fingerprint. This reconstruction can be done at different levels, spanning from assessing the chromatic accessibility that is linked to differential gene expression, to estimating the differential gene expression itself by identifying the spatiotemporal expression pattern of specific gene products (mRNAs and proteins). Traditionally, *in situ* hybridization has been broadly used to detect mRNA molecules within the cells by using labeled antisense RNA probes that hybridize to the specific target. The probe-mRNA hybrid is recognized by an antibody conjugated with specific enzymes that upon administration of their substrate produce either colored compounds or fluorescence.

Nowadays, technologies that allow the capturing of transcriptomes at a single cell resolution and the integration of such mRNA readout data to specific cell types, through spatial transcriptomics, have led to the creation of extensive cell type inventories in various taxa across the evolutionary tree (Sebe-Pedros et al., 2018; Swapna et al., 2018; Cao et al., 2019; Chestnut et al., 2020; Paganos et al., 2021; Qi et al., 2021). The expansion of such inventories reinforced the need to develop reliable *in situ* hybridization protocols as the computationally-predicted cell type need to be validated. Ideally, such protocols should provide high efficiency both in terms of detectability, as well as experimental time, and to be compatible with various organisms.

In this study, we developed and tested a whole-mount fluorescent *in situ* hybridization (FISH) method, with broad applicability on many marine embryos and larvae. This protocol allows an overnight hybridization and the whole procedure can be completed within 2 or 3 days, depending on the number of probes used (single or double *in situ* hybridization). Moreover, the main advantage of this protocol is that, with minor methodological adaptions (from fixation to the actual *in situ* hybridization procedure), it is compatible with many marine organisms. Here we show FISH data obtained in mollusks, echinoderms, tunicates and cephalochordates.

## Materials and Methods

### Probe Synthesis

Probe synthesis was performed as previously described by: 1) Perillo et al. (2021) for the sea urchin species *Strongylocentrotus purpuratus*, *Strongylocentrotus franciscanus, Lytechinus variegatus*, *Paracentrotus lividus*, *Arbacia lixula*, and the starfish *Patiria miniata;* 2) Annona et al. (2017) for the amphioxus species *Branchiostoma lanceolatum*; 3) D’Aniello et al., 2011 for the sea squirt *Ciona robusta* (previously named *Ciona intestinalis*); 4) Balseiro et al. (2013) for the Mediterranean mussel *Mytilus galloprovincialis*. In summary, probes were synthetized from linearized, cloned and amplified DNA fragments corresponding to each gene of interest that were used as a template for *in vitro* transcription. In the case of digoxigenin and fluorescein labelled probes, labeling was carried out during the *in vitro* transcription according to the manifacturers’ quidelines (Roche), while for DNP labelled probes, non-labelled RNA was synthetized and was labelled post-transcriptionally, according to the manifacturer’s instructions (Mirus corporation). The gene makers as well as the species in which each was used are listed in Table 1.

**TABLE 1:** List of gene markers used to test uFISH protocol in mollusks, echinoderms, tunicates and cephalochordates.

Gene symbol	Gene Name	Species
Act	Actin	*M. galloprovincialis*
Cdx	Caudal type homeobox	*S. purpuratus; P. miniata*
Fbsl	Fibrosurfin-like	*S. purpuratus*
Fgf9/16/20	Fibroblast growth factor 9/16/20	*S. franciscanus*
FoxE	Forkhead box E	*B. lanceolatum*
Hnf6	Hepatocyte nuclear factor 6	*C. robusta*
ManRC1a	Macrophage mannose receptor 1	*A. lixula*
Pax6	Paired box 6	*P. lividus*
Pdx1	Pancreas/duodenum Homeobox 1	*S. purpuratus*
Spec1	Calcium-binding SPEC 1A	*S. purpuratus*
SynB	Synaptotagmin B	*L. variegatus*
Vasa	ATP-dependent RNA helicase vasa	*S. purpuratus*

### Animal Collection

Adult individuals of the sea urchin species *P. livdus* and *A. lixula* were collected from the Gulf of Naples (Italy); adult *C. robusta* individuals were collected from the Gulf of Taranto (Italy); the sea urchin *S. purpuratus* and starfish *P. miniata* were collected from the Gulf of Santa Catalina (CA, United States) and distributed by Patrick Leahy (Kerckhoff Marine Laboratory, California Institute of Technology, Pasadena, CA, United States). All the aforementioned species were housed in circulating aquaria at Stazione Zoologica Anton Dohrn (Naples, Italy). Adult individuals of *M. galloprovincialis* were purchased from Irsvem Srl, a commercial shellfish farm (Naples, Italy) and were used immediately for spawning. *B. lanceolatum* embryos and larvae were reared from animals collected in Argelès-sur-mer (France) and spawned at Observatoire Océanologique de Banyuls-sur-Mer (France). The sea urchin *S. franciscanus* larvae were collected from adult individuals collected from the Gulf of Santa Catalina (CA, United States) and spawned at Kerckhoff Marine Laboratory.

### *In vitro* Fertilization of Gametes and Rearing of Embryos and Larvae

**Sea urchin:** Gametes were obtained by vigorous shaking of the adult individuals: zygotes were formed after the fertilization of eggs with approximately 1:1,000 dry sperm diluted in filtered sea water (FSW) and embryos were let to grow according to their species salinity and temperature biological needs. In detail, *A. lixula* and *P. lividus* larvae were cultured at 18°C in Mediterranean FSW, *L. variegatus* larvae at 23°C in artificial sea water (ASW) and *S. purpuratus* as well as *S. franciscanus* at 15°C in Mediterranean FSW diluted 9:1 with deionized water and Pacific Ocean FSW respectively. *L. variegatus* larvae were a gift from Dr. Margherita Perillo.

**Starfish:** Gametes from the batstar *P. miniata* male and female individuals were collected by performing a 4 mm diameter incision on one side of each animal’s arm. A piece of each gonad was extracted and placed in calcium and magnesium-free ASW. In case of female gametes, immature eggs were treated with 10 μM 1-Methyladenine in Mediterranean FSW for 1 hour until the germinal vesicle (GV) disappears, indication of their maturity. Mature eggs were then fertilized with approximately 1:1,000 dry sperm diluted in FSW and zygotes were let to grow at 15°C in Mediterranean FSW diluted 9:1 with deionized water.

**Sea squirt:** Gametes from *C. robusta* male and female individuals were taken separately for *in vitro* fertilization followed by chemical dechorionation and fertilization (D'Aniello, 2009). Embryos were let to grow until the stage of interest at 18°C in Mediterranean FSW.

**Mussel:** Sexually mature specimens of *M. galloprovincialis* were mechanically stimulated for the spawning. Approximately, 20–30 mussels were placed in a tank with Mediterranean FSW at 18°C and spread to easily monitor the spawning. When mussels began to spawn, each individual was washed and then transferred into a Becker containing 200 ml of Mediterranean FSW to isolate males and females. Mature eggs were fertilized with an eggs/sperm ratio 1:15 in a volume of 50 ml. The resulting zygotes were let to grow at 18°C in Mediterranean FSW until the developmental stage of interest.

**Amphioxus:** *B. lanceolatum* gametes were obtained during late spring/early summer period and the spawning was induced by applying thermal shock (Fuentes et al., 2007). Mature gametes were collected and directly used for the *in vitro* fertilization. Embryos were let to grow at 18°C in Mediterranean FSW.

### Fixation

Fixation is an essential step and needs to be performed properly to ensure the mRNA integrity of the samples. All embryos and larvae were fixed in 4% PFA in MOPS Buffer (4% paraformaldehyde, 0.1 M MOPS pH 7, 0.5 M NaCl in DEPC MQ water). Fixation was carried out either for 1 h at room temperature (RT) or overnight at 4°C (both types of fixation gave similar results in all the mentioned species). After fixation was complete, specimens were washed 3–5 times with MOPS buffer (0.1 M MOPS pH 7, 0.5 M NaCl and 0.1% Tween-20 in nuclease-free water) and then gradually dehydrated by passing them through 50%, 60% and finally 70% ice cold ethanol. Samples in 70% ethanol were kept at −20°C until use. The dehydration process can be skipped in case the samples will be used immediately.

### Whole-Mount Fluorescent *in situ* Hybridization (FISH)

***Day 1*:** The first day of the FISH procedure includes the rehydration of the specimen and their incubation with the specific antisense RNA probes. **Rehydration:** Embryos/larvae stored in 70% ethanol were placed in 1.5 ml Eppendorf tubes and were gradually rehydrated in MOPS buffer and washed 3–5 times at RT. The duration of each wash was 15 min. In the case of *Branchiostoma lanceolatum*, FISH was also performed on embryos and larvae that were treated with proteinase K (5 μg/ml) to facilitate probe penetration as previously described in Annona et al., 2017. **Pre-hybridization:** After rehydration was complete, MOPS buffer was replaced by hybridization buffer [50% formamide, 0.1 M MOPS pH 7, 0.5 M NaCl and 0.1% Tween-20, 1 mg/ml Bovine serum albumin (BSA) in nuclease-free water] that does not contain any probes and the specimen were kept at 65°C for 3 h. Within this time interval, hybridization buffer was exchanged once to ensure there are no MOPS buffer residues and that the specimens are incubated in the proper hybridization buffer concentration. **Hybridization:** After the pre-hybridization step, specimens were then incubated overnight at 65°C in the hybridization buffer containing the probe. The probe/hybridization buffer solution was preheated for 5–10 min at 70°C followed by 5 min on ice, a step necessary to denature the probe molecules. The antisense RNA probes (labelled with either digoxigenin, fluorescein or dinitrophenol) were used at a final concentration of 0.3–0.5 ng/μl.

***Day 2:*** The second day of the FISH procedure includes all the steps ranging from removing the non-hybridized probe residuals and incubating with the appropriate antibody conjugated with horseradish peroxidase (HRP) to signal detection and imaging. **Post-hybridization:** The probe/hybridization buffer solution was replaced with hybridization buffer that does not contain probes and specimens were incubated for 1 h and 30 min at 65°C. This wash step was performed two times in total. Next, the embryos/larvae were washed 3–5 times with MOPS buffer at RT. The interval of each wash was 15 min. **Blocking and antibody incubation:** Specimens were incubated in the TSA Plus blocking reagent (0.5% blocking reagent in MOPS buffer) for 30 min at RT. The TSA Plus blocking reagent can also be substituted by a homemade blocking solution containing 1 mg/ml BSA and 4% Sheep serum in MOPS buffer. During the blocking step, the antibody solution (1:1,000 of either anti-DIG or anti-DNP or anti-FLUO HRP-conjugated in blocking solution depending on how the probe was labelled) was also let to incubate for 30 min at RT. Next, the blocking solution was replaced with the antibody-containing solution and incubated for 1 h at 37°C. The antibody incubation is followed by MOPS buffer washes (3–5 times) to ensure the removal of the unbound antibody molecules. The duration of each wash was 15 min. **Staining:** The specimens were incubated with 0.005% H_2_O_2_ in TBS 1x (amplification buffer) for 15 min. From this step and onwards specimens must always be kept in the dark. Next, amplification buffer was replaced by the cyanine-containing solution (Cy3 or Cy5 diluted 1:400 in amplification buffer) and left to incubate for 15 min. Samples were then washed for 15 min, 3–5 times, with MOPS buffer to remove the cyanine residuals. At this point, MOPS buffer containing DAPI (final concentration of 1 μg/ml) was added and the specimens were ready to be observed, **Double FISH**: In case of a double FISH, the staining step performed as described, is followed by a 30 min incubation of the samples with a solution containing 1% H_2_O_2_ in MOPS buffer that allows the inactivation of the HRP-conjugate to the antibody detecting the first probe. Thereafter, a second round of “blocking and antibody incubation” and of “staining” are provided. For the second staining it is important to use the appropriate antibody according to the labeling of the second probe and a Cyanine different from the one used in the first staining to avoid the false co-localization of the two probes used. For both single and double FISH, the last MOPS buffer wash of the “staining” step was replaced with MOPS buffer containing DAPI in a final concentration of 1 μg/ml and samples were mounted observation with a Zeiss LSM 700 confocal microscope. A protocol format of the FISH procedure can be found in Supplementary Material S1.

## Results

Reconstruction of gene expression patterns is of high importance for understanding the molecular fingerprint of distinct cell types and for gaining insight into their functions. In this study we developed a fast fluorescent *in situ* hybridization protocol that we then tested on a variety of marine organisms.

This protocol is the result of modifications of a previous one developed by our group and used in echinoderms (two sea urchin and one starfish species) (Perillo et al., 2021). These modifications are regarding the formamide content of the hybridization buffer, which percentage was lowered (from 70% in Perillo et al., 2021 to 50% in the present work) and the subsequent increase of the hybridization temperature (from 50°C in Perillo al., to 65°C here) that led to the acceleration of the whole procedure from approximately 10 days to 2–3 days in total. In addition, one of the most exciting outcomes of the FISH protocol was that we applied it not only to additional echinoderms species, but also to other species representatives of the mollusks, tunicates and cephalochordates.

In detail, here we present FISH examples for five sea urchin species (*S. purpuratus*, *S. franciscanus*, *L. variegatus*, *P. lividus* and *A. lixula*), the starfish *P. miniata*, the mollusk *M. galloprovincialis*, the tunicate *C. robusta* and the cephalochordate *B. lanceolatum*.

### Echinoderms

**FIGURE 1:**
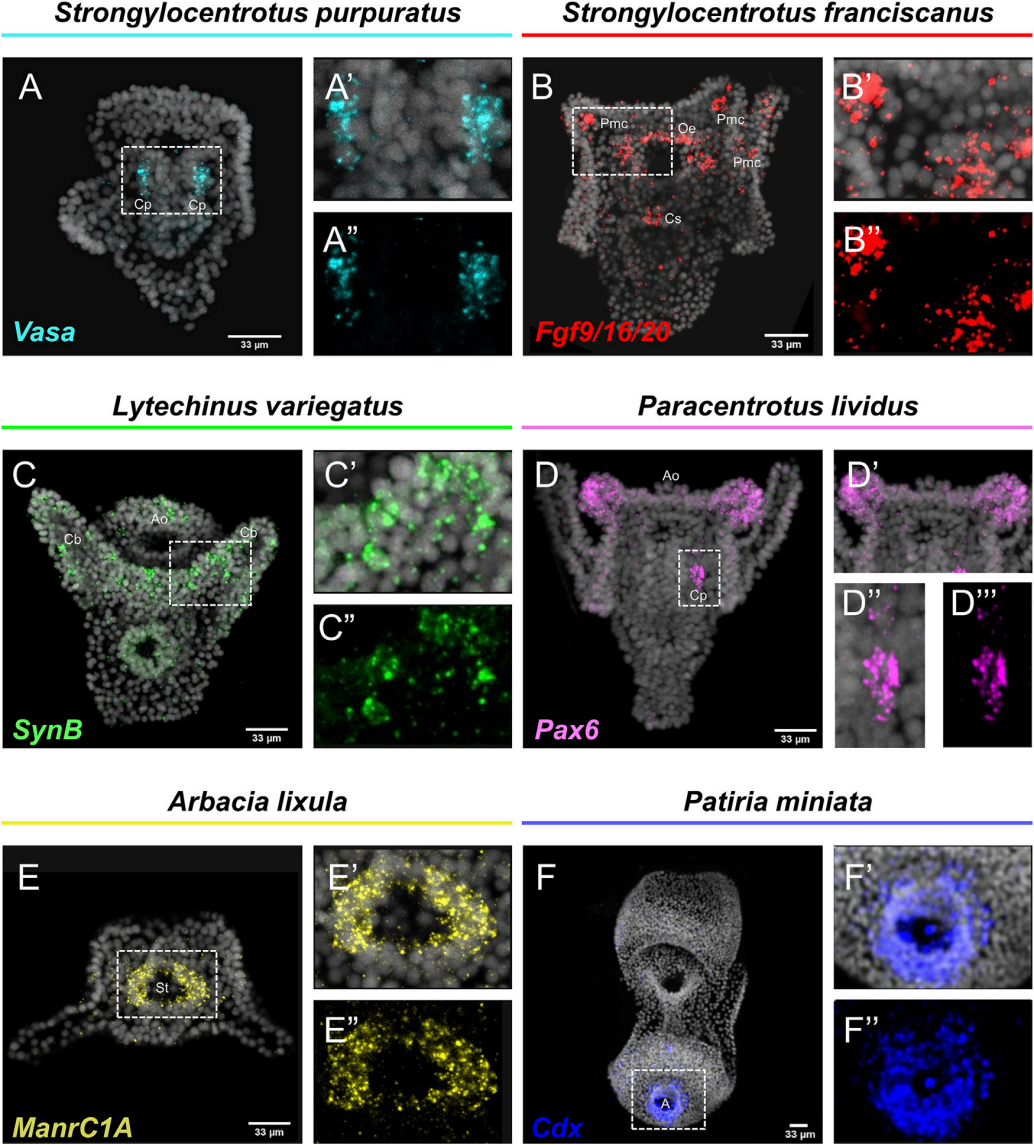
Expression patterns of known gene markers in echinoderm representatives through FISH. FISH of *S. purpuratus* larva with antisense probe for *Vasa* **(A–A'')**, *S. franciscanus* larva with antisense probe for *Fgf9/16/20* **(B–B'')**, *L. variegatus* larva with antisense probe for *SynB* **(C–C'')**, *P. lividus* larva with antisense probe for *Pax6* **(D–D'')**, *A. lixula* with antisense probe for *ManrC1A* **(E–E'')** and *P. miniata* larva with antisense probe for *Cdx* **(F–F'')**. Nuclei are stained with DAPI (in grey). All images are stacks of merged confocal Z sections. A, Anus; Ao, Apical organ; Cb, Ciliary band; Cp, Coelomic pouches; Cs, Cardiac sphincter; Oe, Oral ectoderm; Pmc, Primary mesenchyme cell; St, Stomach.

**FIGURE 2:**
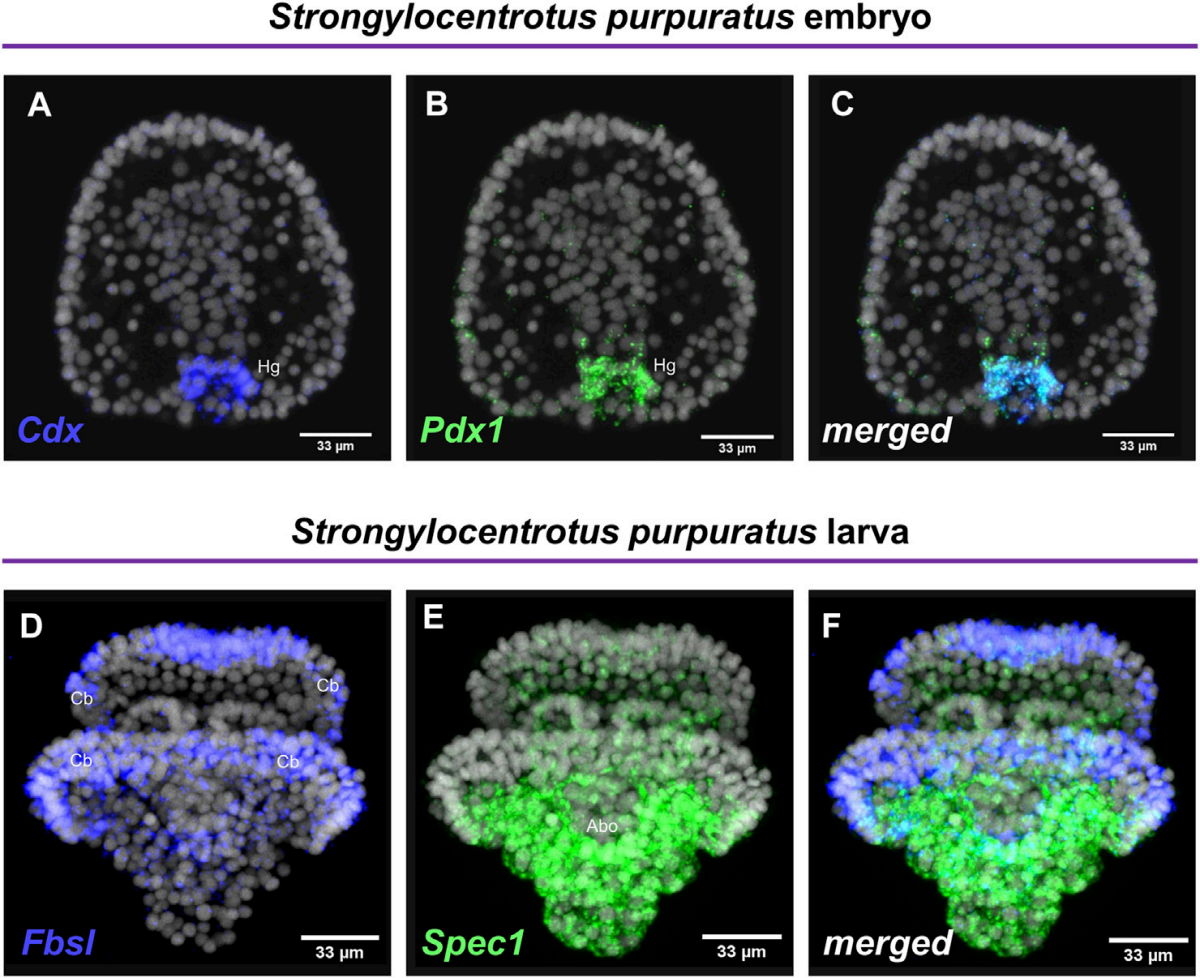
Examples of double FISH. Double FISH of *S. purpuratus* embryos at gastrula stage using antisense RNA probes against *Cdx* **(A)** and *Pdx1* **(B)**. The overlay of the two channels is shown in panel **(C)**. Double FISH of *S. purpuratus* pluteus larvae using antisense RNA probes against *Fbsl* **(D)** and *Spec1* **(E)**. The overlay of the two channels is shown in panel **(F)**. Nuclei are labelled with DAPI (in grey). All images are stacks of merged confocal Z sections. Abo, Aboral ectoderm; Cb, Ciliary band; Hg, Hindgut.

Embryos and larvae from six echinoderm species were used to test the efficiency of the FISH protocol, for which we used antisense probes designed against gene markers known to label distinct cell types. In detail, FISH for the small micromere descendant cells gene marker *Vasa* (Juliano et al., 2006; Paganos et al., 2021), resulted in the detection of *Vasa* transcripts accumulated in the coelomic pouches of the *S. purpuratus* pluteus larva, structures that are formed mainly by small micromere descendant cells (Figures 1A–A″). Transcripts for the Fgf signaling ligand, *Fgf9/16/20,* were detected in the skeletogenic primary mesenchyme cells (Pmc) located in the oral and post-oral arms as well as oral ectoderm and cardiac sphincter cells of the *S. franciscanus* pluteus larva (Figures 1B–B″) domains known to be *Fgf9/16/20* positive in the larvae of other sea urchin species (Röttinger et al., 2008; Paganos et al., 2021). Using a specific antisense probe for the sea urchin pan-neuronal marker *SynB* we were able to detect transcripts present in scattered neurons all across the ciliary band and the apical plate of the *L. variegatu*s sea urchin larva (Figures 1C–C″), in agreement with previous studies (Burke et al., 2006; McClay et al., 2018; Tsironis et al., 2021). Moreover, FISH for the *Pax6* gene in the *P. lividus* larva was able to fully reconstruct its expression domains including two bilaterally symmetric ectodermal patches that correspond to the oral arms of the larva and the coelomic pouch domains (Figures 1D–D″), in agreement with published FISH data (Tsironis et al., 2021). Furthermore, FISH for the midgut specific gene marker *ManrC1a* revealed the presence of its transcripts in a specific domain of the digestive tract of the *A. lixula* larva already described for other species (Annunziata et al., 2014) (Figures 1E–E″). Lastly, using the antisense probe against the parahox gene *Cdx* we were able to reconstruct the same expression profile of this gene in the posterior gut of the *P. miniata* starfish larva (Figures 1F–F″), as previously shown by our group using a different FISH protocol (Annunziata et al., 2013). Finally, to assess whether the accelerated hybridization process had any impact on FISH performed with more than one probes, we used this protocol to performed double FISH on *S. purpuratus* embryos and larvae. Strikingly, our data suggest that the hybridization conditions of the FISH do not affect the ability of more than one probe to hybridize or lower the efficiency of the hybridization. Examples of this can be found in Figure 2 in which we demonstrate that genes known to be co-localized, as shown by different hybridization conditions in the past, are still found to be co-localized through FISH. In detail, *Cdx* and *Pdx1* transcripts co-localize in cells located in the hindgut region of the gastrula embryo (Figures 2A–C) similar to what has been previously demonstrated (Cole et al., 2009). Moreover, we were able to clearly discriminate the expression of genes known to never co-localize as in the case of *Fbsl* and *Spec1*, maker genes of the ciliary band and of the aboral ectoderm respectively (Paganos et al., 2021). Using the FISH protocol, *Fbsl* and *Spec1* showed distinct accumulation of transcripts (Figures 2D–F) in the same domains we expected from the already published expression pattern data.

### Mollusk, Tunicates and Cephalochordates

Once we demonstrated that this fast FISH protocol is able to reconstruct the same expression domains of well-known echinoderm cell type markers, we set out to investigate whether the FISH protocol is also applicable to other marine organisms.

**FIGURE 3:**
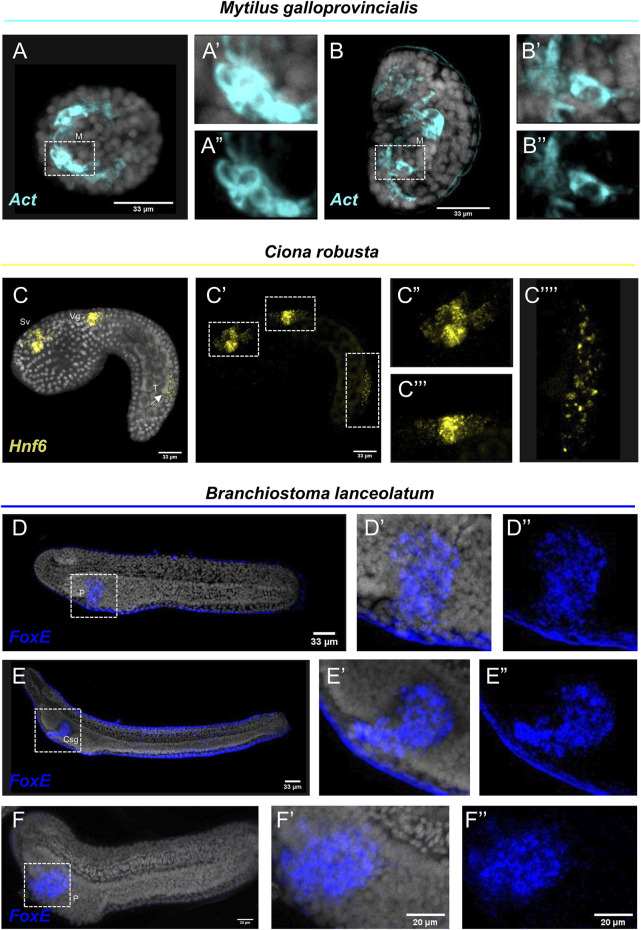
Expression patterns of known gene markers in mollusk, tunicate and cephalochodate representatives through FISH. FISH of *M. galloprovincialis* embryos **(A–A'')** and larvae **(B–B'')** with antisense probe for *Actin*; *C. robusta* embryo with antisense probe for *Hnf6* **(C–C'')**; *B. lanceolatum* embryo **(D–D'')** and larva **(E–E'')** not treated and treated with Proteinase K **(F–F'')** with antisense probe for *FoxE*. Nuclei are labelled with DAPI (in grey). All images are stacks of merged confocal Z sections. Csg, club-shaped gland; M, Muscle; P, Pharyngeal endoderm; Vg, Visceral ganglion; Sv, Sensory vesicle; T, Tail. Arrow indicates positive cells in the posterior neural tube.

Interestingly, FISH for the *M. galloprovincialis Actin* gene resulted in the detection of transcripts in bilaterally symmetric populations of muscles in the embryo (Figures 3A–A″) and in a larger population of muscles on the interior of the shell at the veliger larva (Figures 3B–B″). Such expression patterns are in agreement with previous studies using chromogenic *in situ* hybridization protocols (Balseiro et al., 2013).

*C. robusta* embryo was used as a representative of the tunicate clade. FISH using an antisense probe against *hnf6,* a member of the Cut homeobox gene family, fully reconstructed its previously described expression domains (D'Aniello et al., 2011) along the anterior-posterior axis including precursors of the sensory vesicle, of the visceral ganglion and distinct cell populations of the posterior neural tube (Figures 3C–C‴). Interestingly, the *hnf6* signal localized in the tail of the *C. robusta* embryo (Figure 3C‴′) is an expression domain that, according to the authors who first described it, is hard to detect as it appears only after prolonging the staining time (Pezzotti et al., 2014). Nevertheless, this is another piece of evidence supporting that our protocol can easily and clearly reconstruct complex expression patterns in *C. robusta* embryos.

Last but not least, we tested the efficiency of the FISH protocol on a representative of the cephalochordate clade, the amphioxus species *Branchiostoma lanceolatum*. *FoxE* is a transcription factor known to be expressed during the amphioxus embryogenesis from neurula until the larva stage. It has been previously demonstrated by chromogenic *in situ* hybridization, that *FoxE* is specifically expressed ventrally on the right side of pharyngeal endoderm at the late neurula stage, while at larva stage its expression is detected in the club-shaped gland (Aldea et al., 2015). FISH for the *FoxE* gene with our protocol shows accumulation of transcripts in the aforementioned domains at both developmental stages (Figures 3D–E″), highlighting that FISH is also applicable on this marine species. However, among all the species tested, *B. lanceolatum* specimens displayed the highest degree of background to signal ratio, which could potentially indicate probe trapping in the *epidermis*. To account for this, we also performed our FISH protocol on specimen that after rehydration were treated with Proteinase K (Figures 3F–F″) that is known to improve probe penetration in this taxon. Indeed, while the specificity of the signal between treated and not treated specimens is unaltered, the background around the *epidermis* was almost completely abolished.

## Discussion

**FIGURE 4:**
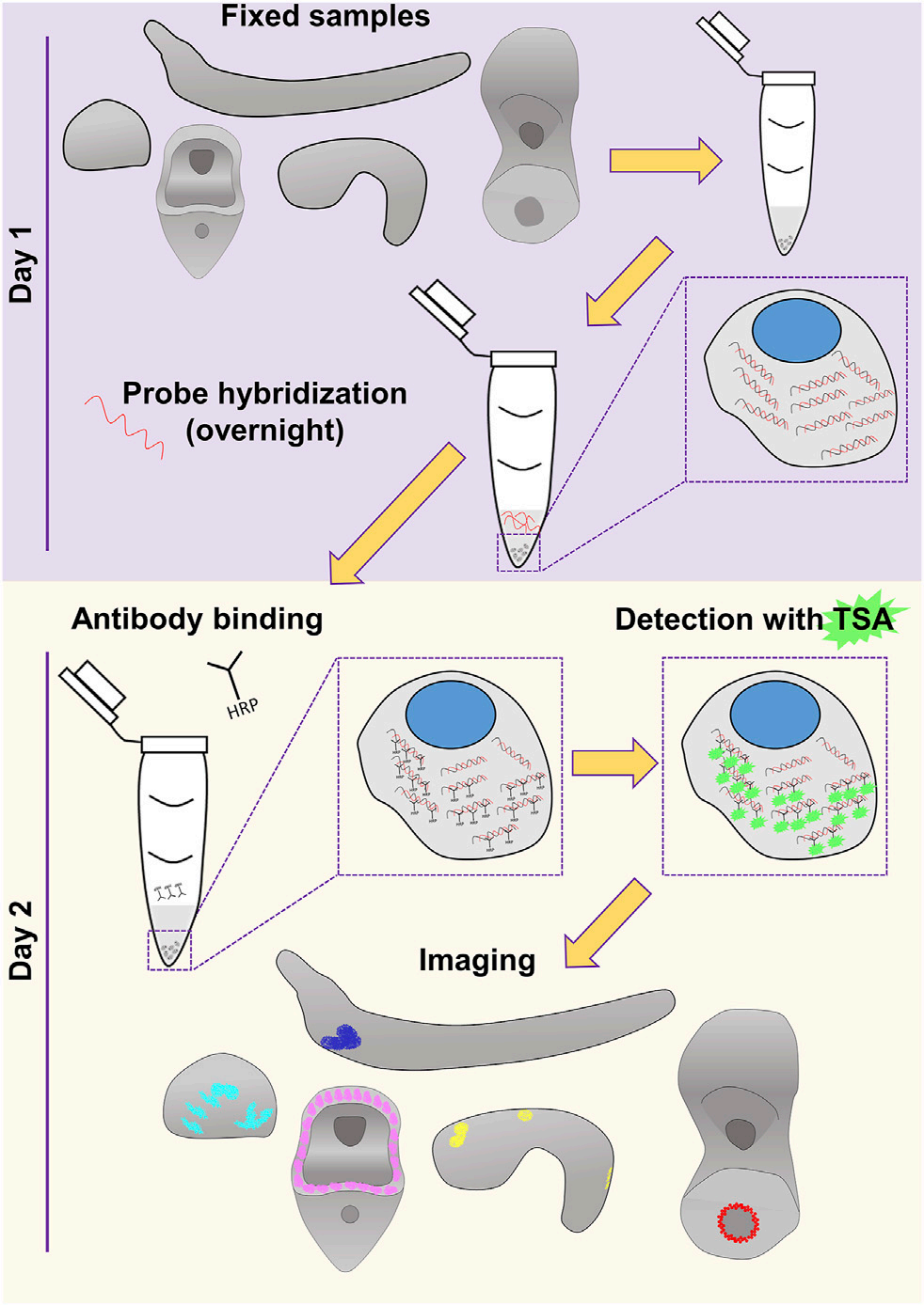
Overview of the FISH procedure described in this study. Schematics of the embryos and larvae used to test the protocol are represented in gray. Color code of the staining corresponds to what used in Figures 1–3.

Nowadays, extensive cell type inventories are being generated for taxa across the evolutionary tree in an attempt to understand the molecular fingerprint, function and evolutionary origins of cell types and thus animals. Current technologies allow the computational reconstruction of cell types either through the use of single cell transcriptomics or spatial transcriptomics. However, the need to verify such computationally based predictions and the challenges to develop reliable molecular biology tools towards this end still remain. This is the main reason why classical methods to visualize gene expression have not gone obsolete and it is highly unlikely they will in the near future. In this work, we provide evidence of our FISH protocol being such a reliable tool that allows the accurate, easy and fast identification of expression domains. An overview of the protocol is depicted in Figure 4.

In the case of echinoderms, FISH dramatically reduces the time required for whole mount FISH procedure from 10 days to only 2–3 days in total, without compromising the quality of the FISH. This feature can be of great importance when considering the higher number of genes that can be analyzed within the additional time offered by FISH. Regarding the mussel *M. galloprovincialis,* there are only a few for whole-mount ISH protocols available, mostly for chromogenic *in situ* hybridization (Murray et al., 2003; Balseiro et al., 2013). Surprisingly, this is the first study to use whole-mount FISH to detect mRNAs present during *M. galloprovincialis* embryonic development. Concerning the tunicate and cephalochordate representatives, *C. robusta* and *B. lanceolatum,* respectively, FISH might be an easier alternative utilizing less reagents in terms of variety and potentially requiring less experimental time when compared to existing protocols (Yu and Holland, 2009; D’Aniello et al., 2011; Annona et al., 2017). Nonetheless, depending on the intrinsic properties of the sample, minor adaptations could be introduced as in the case of *C. robusta* and *B. lanceolatum* that chemical dechorionation and Proteinase K treatment respectively, are advised to obtain a reduced background to signal ratio.

Overall, it is important that the experimental conditions are kept as similar as possible for most comparisons with a biological significance, taking into consideration of the intrinsic properties of the samples to be analyzed. Towards this end, we believe that using a universal *in situ* protocol for marine organisms, similar to how we universally use other molecular biology techniques (such as PCR, RNA-seq) could potentially ease cross-species comparisons by removing the heterogeneity in how samples are processed. Overall, our FISH protocol has the potential to be an extremely useful resource for Evo-Devo community by allowing FISH to be performed in species previously thought impossible. Whether this protocol could also be applied on non-marine species is still an open question, although we believe that adaptation of the solutions used to the osmotic needs of terrestrial or freshwater organisms should be sufficient to ensure its functionality also to such specimens. Nonetheless, further information and experimentation are needed to achieve this goal and thus testing of this protocol in additional taxa is required.

## Data Availability

The original contributions presented in the study are included in the article/Supplementary Material, further inquiries can be directed to the corresponding authors.
