# Supplementary material for: FISH for All: A Fast and Efficient Fluorescent In situ Hybridization (FISH) Protocol for Marine Embryos and Larvae
Source: Front Physiol. 2022 Apr 19;13:878062. doi: 10.3389/fphys.2022.878062 (PMC9062072; doi:10.3389/fphys.2022.878062)
Supplement: Supplementary file 1 [file DataSheet1.DOCX]

**FISH for all: A fast and efficient Fluorescent *In Situ* Hybridization (FISH) protocol for marine embryos and larvae**

Periklis Paganos*, Filomena Caccavale, Claudia La Vecchia, Enrico D’Aniello, Salvatore D’Aniello and Maria Ina Arnone*****

Department of Biology and Evolution of Marine Organisms, Stazione Zoologica Anton Dohrn, Villa Comunale 80121, Naples, Italy

*** Correspondence:** [miarnone@szn.it](mailto:miarnone@szn.it); [periklis.paganos@szn.it](mailto:periklis.paganos@szn.it)

**Day 1**

**Rehydration**

1. Place embryos/larvae stored in 70% ethanol in 1.5 ml Eppendorf tubes and wash with MOPS buffer (approximately 3-5 times) at RT. The duration of each wash is 15 min.

**Pre-hybridization**

1. MOPS buffer is replaced by hybridization buffer that does not contain any probes and the specimen are kept at 65°C for 3 h. Pre-hybridization solution is exchanged twice.

**Hybridization**

1. The probe/hybridization buffer solution is preheated for 5-10 min at 70°C followed by 5 min on ice, a step necessary to denature the probe molecules. The antisense RNA probes (labelled with either digoxigenin, fluorescein or dinitrophenol) are used at a final concentration of 0.3-0.5 ng/μl and samples are left in hybridization buffer solution at 65°C O/N.

**Day 2**

**Post-hybridization**

1. The probe/hybridization buffer solution is replaced with hybridization buffer that does not contain probes and specimens are incubated for 1 h and 30 min at 65°C.
2. Repeat wash step and let specimens to incubate for an additional 1 h and 30 min.
3. Embryos/larvae are washed 3-5 times with MOPS buffer at RT. The interval of each wash is 15 min.

**Blocking and antibody incubation**

1. Specimens are incubated in the TSA Plus blocking reagent (0.5% blocking reagent in MOPS buffer) for 30 min at RT. During the blocking step, the antibody solution (1:1000 of either anti-DIG or anti-DNP or anti-FLUO HRP-conjugated in blocking solution depending on how the probe was labelled) is also let to incubate for 30 min at RT.
2. Replace blocking solution with the antibody-containing solution and incubate for 1 h at 37°C.
3. Remove antibody-containing solution and wash with MOPS buffer 3-5 times to ensure the removal of the unbound antibody molecules. The duration of each wash is 15 min.

**Staining**

1. The specimens are incubated with 0.005% H_2_O_2_ in TBS 1x (amplification buffer) for 15 min. (From this step and onwards specimens must always be kept in the dark).
2. Replace amplification buffer with cyanine-containing solution (Cy3 or Cy5 diluted 1:400 in amplification buffer) and incubate for 15 min.
3. Remove cyanine-containing solution and wash with MOPS buffer for 15 min (3-5 times), to remove the cyanine residuals.
4. In the case of single FISH add MOPS buffer containing DAPI (final concentration of 1 µg/ml) and mount samples for observation.

**Double FISH**

1. Remove MOPS buffer, add 1% H_2_O_2_ in MOPS buffer and let to incubate for 30 min. (This step allows the inactivation of the HRP-conjugate to the antibody detecting the first probe).
2. Wash with MOPS buffer 3-5 times (15 min each wash).

**Blocking and antibody incubation:**

1. Specimens are incubated in the TSA Plus blocking reagent (0.5% blocking reagent in MOPS buffer) for 30 min at RT. Once more, the antibody solution (1:1000 of either anti-DIG or anti-DNP or anti-FLUO HRP-conjugated in blocking solution depending on how the second probe was labelled) is also let to incubate for 30 min at RT.
2. Replace blocking solution with the antibody-containing solution and incubate for 1 h at 37°C.
3. Remove antibody-containing solution and wash with MOPS buffer 3-5 times to ensure the removal of the unbound antibody molecules. The duration of each wash is 15 min.

**Staining**

1. The specimens are incubated with 0.005% H_2_O_2_ in TBS 1x (amplification buffer) for 15 min. (From this step and onwards specimens must always be kept in the dark)
2. Replace amplification buffer with cyanine-containing solution (Cy3 or Cy5 diluted 1:400 in amplification buffer) and incubate for 15 min. You **must** choose a different Cyanine to the one you used in step 10.
3. Remove cyanine-containing solution and wash with MOPS buffer for 15 min (3-5 times), to remove the cyanine residuals.
4. Replace MOPS buffer with MOPS buffer containing DAPI (final concentration of 1 μg/ml) and mount samples for observation.

**Notes/Troubleshooting**

1. To ensure the preservation of the riboprobes, all the solutions are prepared using DEPC-treated water or nuclease free water. When using the riboprobes to make the appropriate dilution in hybridization buffer keep the riboprobes on ice to avoid RNA degradation.
2. When exchanging buffer solutions always keep the embryos in at least twice the volume of the embryo/larvae pellet to ensure they do not dry out.
3. Always use gentle pipetting to ensure the structure of the samples will remain intact.
4. Avoid using too many embryos/larvae since this will affect the hybridization efficiency.
5. The volume of each wash should be at least 10 times the embryo/larva pellet. (i.e. for a 20 μl embryo/larva pellet wash with at least 200 μl of each solution).
6. For double FISH add both riboprobes in the same hybridization solution (DIG and or DNP and or FLUO probes).
7. From the staining step and onwards always keep tubes covered in aluminum foil to keep them in the dark.
8. The TSA Plus blocking reagent can also be substituted by a homemade blocking solution containing 1 mg/ml BSA and 4% Sheep serum in MOPS buffer.
9. Antibody incubation times can be adjusted from 1 h at 37°C to O/N at 4°C according to the user’s needs.
10. In case there is high background try to either use smaller volumes of conjugated antibodies, less probe concentration or add additional washing steps with MOPS buffer. In case of organisms that have structures that favor probe trapping you can introduce additional steps such as treatment with Proteinase K or similar treatments to remove such structures after the rehydration and before the hybridization process.

**Reagents and solutions list**

1. Fixation solution: 4% paraformaldehyde (Paraformaldehyde, Electron Microscopy Sciences, cat # 15710, Paraformaldehyde 16% solution), 0.1M MOPS pH 7, 0.5M NaCl 0.1%, in nuclease free water.
2. MOPS buffer: 0.1M MOPS pH7, 0.5M NaCl, 0.1% Tween-20 in nuclease free water.
3. Hybridization buffer: 50% formamide (Sigma-Aldrich cat# F9037), 0.1 M MOPS pH 7, 0.5 M NaCl and 0.1% Tween-20, 1 mg/ml Bovine serum albumin (BSA) in nuclease-free water.
4. DIG RNA Labeling Mix, Roche cat# 11277073910.
5. Fluorescein RNA Labeling Mix, Roche cat# 11685619910.
6. Label It DNP labeling Kit, Mirus corporation Cat. # MIR 3800.
7. Anti-DIG-POD antibody, Roche cat # 11207733.
8. Anti-FLUO-POD antibody, Roche cat# 11426346910.
9. Anti-DNP-HRP cat # NEL747B.
10. TSA Plus Cyanine 3 Kit, Akoya Biosciences cat # NEL744001KT.
11. TSA Plus Cyanine 5 Kit, Akoya Biosciences cat # NEL745001KT.
12. TSA Plus Fluorescence Palette Kit (Blocking reagent, amplification reagents, amplification diluent) cat# NEL760001KT. The kit provides the blocking reagent as a powder that is used to make 0.5% blocking reagent in MOPS buffer.
